# Dental manifestations and treatment of hypophosphatemic rickets: A case report and review of literature

**DOI:** 10.1038/s41405-023-00129-9

**Published:** 2023-01-30

**Authors:** Xinyang Jin, Yuedan Xu, Wei Liu, Zhiwei Shi, Yi Sun, Xinni Pan, Ling Zhang, Baiping Fu

**Affiliations:** 1 Department of Stomatology, The Fourth Affiliated Hospital, Zhejiang University School of Medicine, Yiwu, China; 2 Department of Prosthodontics, Stomatology Hospital, School of Stomatology, Zhejiang University School of Medicine, Zhejiang Provincial Clinical Research Center for Oral Diseases, Key Laboratory of Oral Biomedical Research of Zhejiang Province, Cancer Center of Zhejiang University, Engineering Research Center of Oral Biomaterials and Devices of Zhejiang Province, Hangzhou, China

**Keywords:** Oral diseases, Dentistry

## Abstract

**Background:**

The treatment and management of patients suffering from hypophosphatemic rickets (HR) remain a major challenge for dental practitioners and affected patients.

**Objectives:**

To report a case of HR presenting with specific dental findings and to review the dental manifestations and treatment of HR patients.

**Methods:**

*Case*: A 32-year-old male presented with multiple dental abscesses and short stature. A thorough history was taken followed by clinical oral examination, and relevant radiological investigation was done. *Literature research*: In 2020, electronic literature searches were carried out in PubMed and complemented by a careful assessment of the reference lists of the identified relevant papers. Articles and reports fulfilled the inclusion criteria: indexed reviews, case series and case reports in English and restricted to human studies were considered.

**Results:**

The intraoral examination revealed multiple dental abscesses and general periodontal disease; the radiographic examination showed poorly defined lamina dura, large pulp chambers and periapical lesions. Based on the contents of the 43 articles identified in the search, the current knowledge of dental manifestations, treatment and management of HR was summarized.

**Conclusions:**

As HR is a multisystem disease, multidisciplinary care is needed. By summarizing current evidences, we proposed an evidence-based dental management and provided recommendations on diagnosis and treatment of the disease.

It is of profound clinical significance to acquire knowledge of the dental manifestations and provide optimal treatment options for patients.

## Introduction

Hypophosphatemic rickets (HR) is a group of hereditary metabolic bone diseases caused by renal phosphate wasting, which has no response of high doses of vitamin D [1]. HR is characterized by short stature, delayed walking, bow legs, bone/joint pain, spontaneous dental abscess, hypophosphatemia, and inappropriately normal serum 1,25(OH)_2_D level [2]. Genetic defects in factors necessary for phosphate handling is the main cause of phosphate wasting [3]. HR can be mainly divided into three types, including X-linked hypophosphatemic rickets (XLHR) due to a loss function of phosphate regulating gene with homologies to endopeptidases on the X chromosome (PHEX) [4], autosomal dominant hypophosphatemic rickets (ADHR) caused by mutation in the fibroblast growth factor 23 (FGF23) gene [5] and autosomal recessive hypophosphatemic rickets (ARHR) as a result of mutation in dentin matrix protein 1 (DMP1) gene, ecto-nucleotide pyrophos- phatase/phosphodiesterase 1 (ENNP1) gene, or family with sequence similarity 20, member C (FAM20C) [6–8].

XLHR, also named as vitamin D resistant rickets, familial hypophosphatemic rickets, or phosphate diabetes, is the most common form of HR, with an incidence of around 1:20000 [9]. PHEX, mainly expressed in osteocytes and odontoblasts, is located on chromosome Xp22.1 and encodes for an endopeptidase [10]. Inactivating PHEX mutations contribute to the increasing secretion of FGF23 [11]. As a key circulating factor that directs sodium-dependent phosphate transporters in the kidney and intestine, the high level FGF23 results in impaired proximal renal phosphate reabsorption, causing hypophosphatemia and systemic regulation of mineralization [12]. Meanwhile, the PHEX mutations contribute to the local accumulation of mineralization inhibitors such as OPN (osteopontin) and ASARM (the acidic serine- and aspartate-rich motif) peptides at the level of the extracellular matrix [12]. Likewise, the certain genetic mutation emphasizes the importance of FGF23 and DMP1 in the pathogenesis of ADHR and ARHR, respectively [5, 6]. As the same result of phosphate wasting, it is found that different types of HR cause similar, though not identical, clinical and radiographical parameters, which mostly depend on the duration of hypophosphatemia [1].

Early diagnosis of HR is of great significance, since early treatment promotes growth, reduces bone pain and improves dental health [13, 14]. The diagnosis of HR is on the basis of combination of clinical, radiological and biochemical findings. Clinically, patients of HR feature leg bowing, delayed walking, waddling gait, bone/joint pain, short stature and dental abscesses [2]. Especially, any form of leg bowing and widening of the metaphysis acquire a further radiological and biomedical examination [15]. Radiographic manifestations show the long bone deformities and abnormal growth plates with widened and frayed metaphyses [15]. Biomedical criteria for the diagnosis of HR includes serum phosphate below the normal threshold for age, elevated alkaline phosphatase, normal or upper normal parathyroid hormone levels, normal serum calcium, and low urinary calcium excretion [16]. In addition, a family history of the disease is benefit for diagnosis due to an X-linked dominant inheritance pattern of PHEX mutation. Genetic analysis, which identifies mutations in the PHEX gene in about 70% of patients with HR, is the final confirmation [15]. If genetic analysis is not available, elevated plasma levels of intact FGF23 supports the diagnosis [17].

Treatment initiation is recommended as soon as diagnosis is established [17]. Conventional treatment with oral phosphate supplements and active vitamin D (calcitriol or alfacalcidol), has been proposed for decades [3, 18, 19]. This kind of therapy has been shown to result in the positive outcomes of decrease in bone pain, normalization of alkaline phosphatase level, increase in growth velocity, straightening of legs and improving dental mineralization [3, 13, 20, 21]. However, due to the limitations, such as increasing risk of nephrocalcinosis, urolithiasis or hyperparathyroidism, the dose of phosphate supplements and vitamin D analogues should be strictly controlled according to individual situation [22, 23]. Recently, a novel therapy with burosumab, a kind of humanized monoclonal anti-FGF23 antibody, has been approved for the treatment of XLHR patients over 1 year of age in Europe and US, with the function of increasing renal phosphate reabsorption and normalizing serum phosphate [15, 24]. Nevertheless, all of those therapies focus on regulation of systemic serum phosphate, the defects in mineral quality and quantity during formation of mineralized tissues such as tooth and bone due to the local dysfunction of PHEX intend to be treated [11]. Given that, the disease follow-up and treatment management are essential.

For some patients, dental manifestations might be the first symptom diagnosed [25]. Furthermore, early prophylaxis and treatment can improve dental status [14]. Hence, broadening knowledge of various dental manifestations of HR is required to provide timely treatment options, which does help to improve the quality of HR patients’ life [26]. This article aimed at reporting a case of male with HR, reviewing the dental manifestations and treatment of HR patients and providing suggestions of dental management.

## Case report

A 32-year-old male was referred to the Department of Prosthodontics with the complaint of “several abscesses that appeared in the mouth”. The patient was diagnosed with HR at the age of 2, but his parents didn’t have metabolic disorder. Since been diagnosed, he was treated with oral phosphate supplementation, calcitriol and vitamin D2. In addition, he was undergoing pharmaceutical therapy with burosumab at a dose of 60 mg/month by hypodermic injection at the age of 31.

**Fig. 1:**
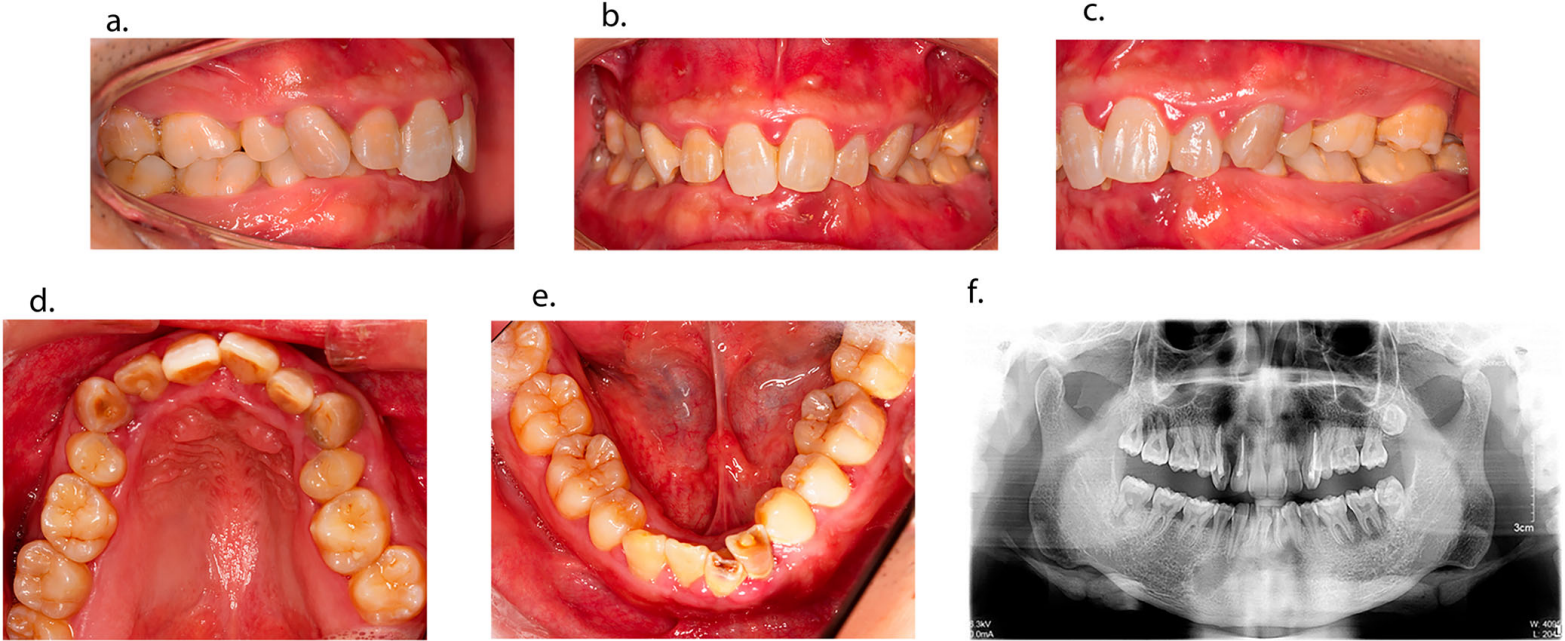
Intraoral views and panoramic radiograph of a 32-year-old male patient with HR. Intraoral views (**a**–**e**) showed multiple fistulas and abscesses, malocclusion, and dental attrition in the left lower incisors; Panoramic radiograph (**f**) revealed enlarged pulp chambers, the radiolucencies at periapical regions of teeth # 12, 13, 17, 22, 23, 32, 36, 37, 41, 42, 43 and 48, and the presence of endodontically treated teeth # 12, 13, 23 and 32.

On physical examination, the patient showed short stature, bow legs, achilles tendinitis and mild hearing loss. The intraoral examination revealed multiple fistulas and abscesses at the periapical regions of maxillary right lateral incisor, mandibular left lateral incisor and first molar, and mandibular right central incisor (Fig. 1a–e). The pulpal vitality test was negative at maxillary right lateral incisor, maxillary right canine and second molar, mandibular left canine, mandibular left lateral incisor and first molar, and mandibular right central incisor and third molar. Periodontal examination revealed the absence of many teeth, and general periodontal disease resulting in some teeth increased probing pocket depth of 10 mm or more. Radiographic examination in Fig. 1f showed poorly defined lamina dura and enlarged pulp chambers and radicular canals. Radiolucencies were detected at periapical regions of maxillary right lateral incisor, maxillary right canine and second molar, mandibular left lateral incisor and canine, mandibular left lateral incisor, mandibular left second and third molar, mandibular right central and lateral incisor, and mandibular right canine and third molar. Several teeth (i.e., maxillary right lateral incisor and canine, maxillary left canine, and mandibular left lateral incisor) had received endodontic treatment before.

## Literature search

A literature search was carried out to identify all cases of HR associated dental manifestations. The PubMed database was searched until 10 March 2020 with the following MESH term: “Rickets, Hypophosphatemic” and a keyword: “dental”. It was supplemented by manual searches in the reference lists of relevant articles. The search retrieved 198 results. Articles and reports including indexed reviews, case series and case reports published in English and in peer-reviewed journals were considered, and restricted to human studies. After screening the title and abstract, 43 relevant full-length articles were included. This search identified about 416 reported cases [12, 14, 21, 25, 27–65], as presented in Table 1.

**Table 1:** Number of reported cases of hypophosphatemic rickets with dental manifestations.

Author	Year	No. of cases	Author	Year	No. of cases
Gigliotti et al. [54]	1971	3	Baroncelli et al. [35]	2006	9
Sauk et al. [27]	1973	1	Chaussain-Miller et al. [14]	2007	7
Cohen et al. [28]	1976	1	Douyere et al. [36]	2009	1
Ainley et al. [29]	1978	1	Souza et al. [37]	2010	14
Nikiforuk et al. [61]	1979	26	Al-Jundi et al. [50]	2010	21
Rakocz et al. [30]	1982	1	Sh AJ et al. [51]	2010	21
Lyles et al. [58]	1985	1	Al-Jundi et al. [46]	2011	1
Bender et al. [53]	1985	50	Ye et al. [47]	2011	10
Abe et al. [59]	1988	3	Beltes et al. [38]	2012	1
Schwartz et al. [49]	1988	18	Rabbani et al. [39]	2012	19
Daley et al. [60]	1990	3	Andersen et al. [45]	2012	52
Hintze et al. [52]	1990	1	Soares et al. [44]	2013	3
Shields et al. [65]	1990	17	Rathore et al. [56]	2013	1
Chadwick et al. [31]	1992	1	Friberg et al. [64]	2013	3
Hillmann et al. [57]	1996	2	Souza et al. [25]	2013	1
Goodman et al. [32]	1998	17	McKee et al. [12]	2013	1
Resnick et al. [63]	1998	1	Cremonesi et al. [40]	2014	10
Murayama et al. [43]	2000	1	Yuan et al. [62]	2015	4
Alexander et al. [55]	2001	1	Ayesha et al. [41]	2016	1
Chaussain-Miller et al. [21]	2003	48	Biosse et al. [48]	2016	34
Pereira et al. [33]	2004	3	Paredes et al. [42]	2017	1
Batra et al. [34]	2006	1	Total cases reported		416

## Dental manifestation

Clinically, recurrent spontaneous dental abscesses both in primary and permanent dentition without carries, periodontal problems, traumatic injuries or restorations are common findings in HR patients [12, 14, 21, 25, 27–44]. Meanwhile, with the increasing age, the number of endodontically affected teeth significantly rises, and the incisors and canines usually get affected prior to molars and premolars [45]. HR patients are more prone to suffer periodontal bone loss than the general population, while the percentage of BOP seems to be similar without commensurate increase [33, 39, 41, 46–48]. Another main dental finding is the smaller dental arches which always accompany with crowded dentition and class III occlusion [29, 46, 49, 50]. Other infrequent dental findings are ectopic permanent canines [34], delayed eruption of both primary and permanent teeth [39], delayed dental development [41, 51] and dental hypersensitivity [52].

Radiologically, both primary and permanent teeth exhibit enlarged pulp chambers with high pulp horns which sometimes extend up to or beyond the dentino-enamel junction [12, 21, 27, 29, 30, 32–34, 37, 39–45, 49, 52–56]. Zones of hypomineralized early forming coronal dentin appear as a “halo” at the dentino-enamel junction around the circumpulpal dentin [52, 55]. Hypoplastic alveolar ridge, reduced radiopacity of lamina dura and varying degree of alveolar bone loss are also radiological characteristics of HR [25, 27, 32, 41, 44, 46–48, 53, 54]. Other common manifestations are thinner enamel and dentin [21, 30, 40, 53], primary tooth resorption [44, 56] and short root [43, 57].

Histopathologically, hypomineralized dentin, featured by a widened predentin with fewer well-defined dentinal tubules and numerous unmerged calcospherites creating interglobular spaces, is commonly seen in teeth of HR patients, while the mantle dentin is unaffected [12, 14, 21, 30–32, 37, 40, 52, 53, 55–60]. The organization and polarization of odontoblasts are also impaired [66]. Enamel alternation, such as numerous crater-shaped depressions and deep microcleavages penetrating into the enamel thickness, can be seen in some patients [21, 32, 35, 37, 39, 49, 58, 61, 62]. Due to the dentin and enamel alternation, bacteria can invade easily from the oral cavity to the dental pulp, causing pulp necrosis in caries-free tooth [39]. Cementum is thinner, with roughly granular with hypomineralized interglobular patterns [48, 67].

## Dental treatment

### Prophylaxis

Preventing bacteria invading dentin and pulp is important to reduce dental abscesses [17]. Stainless steel crown was used to cover the crown of primary molar in the past [30, 55]. However, the limitations of large pulp chamber, high pulp horn, thin enamel and hypomineralized dentin should be taken into strict consideration. Sealing occlusal surfaces of primary and permanent teeth with composite resin is suggested, the properties of which have greatly improved over the last decade [26, 68]. It is worth noting that self-etch adhesive system is recommended to minimize risk of pulp irritation, and the sealing must be repeated every year due to gradual wear of the resin [3]. The early monitored use of topical applications of fluoride is critical for preventing subsequent serious dental infections [33, 36–39, 42]. Tooth attrition is easier to be seen in HR patients for the enamel alternation, so a nightguard acrylic splint is also suggested [53, 55].

### Endodontic treatment

For the endodontically affected teeth, root canal treatment is the conventional choice, while extraction is necessary for those of abscesses spread quickly in jaw bone especially in primary teeth [3]. The procedures of endodontic treatment should try to be sterile. Using sodium hypochlorite to irrigate canals and Ca(OH)_2_ as intracanal medicament for a 10-day interval is recommended, and the determination of working length is preferred to combine electronic apex locators and radiograph [38]. Avoiding any voids and achieving the best possible density of the root canal filling are the goal of the obturation of the root canal system [26]. Meanwhile, the filling of root canal might be suggested using thermoplasticized obturation techniques with a virtually insoluble sealer [26]. Apical curettage procedure and apical barriers with mineral trioxide aggregate might be performed in sever abscesses and open apical foramens, respectively [28, 33].

### Prosthodontic treatment

For some mild or moderate damages, adhesive procedures can be used, but in sever situation, such as dominant enamel fracturing and rapid dental wear, full coverage restoration should be chosen [68]. Regarding to the coronal restoration of endodontic treatment teeth, occlusal coverage with fitted stainless steel crowns is suggested to protect teeth from recurrent infections, however, posts cannot be supported due to the thin dentin [40, 55]. As for the ceramic crown, it is not recommended for teeth with prominent pulp horns, since the preparation costs a greater loss of dentin than metal crown [26]. Therefore, all-ceramic occlusal veneer might be another choice not only as a minimal invasive approach but for aesthetic reason [26].

### Periodontal treatment

Conventional supportive periodontal therapy is of great benefit for HR patients [3]. Hence, twice-yearly visits to perform conventional supportive periodontal therapy aimed to decrease gingival inflammation and suppress periodontal pockets for adults is suggested [17].

### Orthodontic treatment

Although it is confirmed that the periodontium of HR patients is less prone to orthodontic treatment [67], orthodontic treatment is not contraindicated especially for those treated with conventional systemic treatment, and could trigger extensive remodeling of the alveolar bone [68].

### Implant treatment

Implants are acceptable for HR patients and several successful cases have been reported [63, 64]. However, the successful rate is declined in those who are not under conventional treatment, hence, implant surgery is recommended to be performed after 3-6 months of conventional treatment, which should be continued for 6 months following implant surgery [17]. It might be better to prolong the healing time up to 6 months to obtain a good stability [64].

## Dental management

In summary, the lifetime dental management for a particular patient is as followed. (1) Once the diagnosis is established, the conventional treatment should be initiated and last a lifetime if possible [17]. (2) The dental examination should be performed twice a year regularly for adults and children, including dental orthopantomogram, of which the first time is suggested at age 5 [17, 68]. (3) Typical fluoride application and pit and fissure sealing both in primary and permanent teeth should be carried out as soon as acquired [40, 42]. (4) We recommend a thorough dental examination clinically and radiologically searching for all of the enlarged pulp chambers, periapical bone loss and pulp necrosis [17]. (5) In adults, it is suggested to perform conventional supportive periodontal therapy, including periodontal risk assessment as well as supragingival and subgingival debridement twice a year if necessary [17]. (6) If orthodontic and implant treatment are required, it must be based on the premise that conventional therapy is correctly treated [26, 63, 64].

## Discussion

HR is genetic disorders, whose main symptoms are hypomineralized skeleton and dentition [12]. Dental abscesses without caries are observed most frequently in HR, even in some cases as the first symptom diagnosed [25]. In the present report (Fig. 1), the symptoms were enlarged pulp chambers and multiple dental abscesses. The cause of dental abscesses is the enamel alternation and hypomineralized dentin [39]. However, the relationship between HR and hypomineralized dentin remains unclear. In the past, hypophosphatemia was thought to be responsible for dysplastic and poorly mineralized circumpulpal dentin with wide areas of interglobular dentin, which limited growth and fusion of calcospherites [69]. Recent researches have shown that the mineralization induced by human cells is disturbed independently of hypophosphatemia and supported a local role for PHEX, DMP1 or FAM20C in matrix mineralization [11, 70, 71]. This finding might illustrate that conventional therapy improve dental complications but not prevent [35]. Due to different mechanisms of three forms of HR, the article mainly focused on XLHR. Several studies have confirmed that PHEX regulates the mineralization of the extracellular matrix at the local level in mineralized bone and dentin and maintains mineralized matrix homeostasis by cleaving of acidic, non-collagenous SIBLING proteins and peptides of the extracellular matrix such as OPN, MEPE (matrix extracellular phosphoglycoprotein) and ARARM [66, 72, 73]. Furthermore, it is suggested that OPN and MEPE inhibit tooth mineralization through different ways, with OPN acting at the mineralized calcospherites and MEPE at the region of the unmineralized interglobular dentin [66].

From the literature review (Table 1), we can find that the majority of reported patients are females, which seems to indicate a higher HR prevalence in female. However, male patients are usually shown severer dental complications, such as taurodontism [69]. Since most cases are determined by PHEX, an X-linked dominant mutant gene, it might be a gene dose effect [32]. The severity of complications might depend on three factors, including family history, medical history and age. Patients born to affected mother are inclined to bear poorer dental status for primary dentition than those born to healthy mother, due to the lower phosphate and vitamin D obtained from the affected mother during fetal odontogenesis [21]. Nevertheless, a family history contributes to an earlier diagnosis of HR, which might take an earlier treatment for patients, resulting a better outcome for permanent dentition [15]. Although conventional treatment is not able to prevent oral complications, its beneficial effects on dental and periodontal tissues cannot be underestimated [35]. It has already been confirmed that conventional treatment for HR patients improves mineralization of dentin and decreases the incidence of endodontic infection in children [14, 21]. Meanwhile, the benefit of continuing treatment for adults has been proved in permanent teeth and periodontal health recent years [48]. However, the dose of medicine should depend on the age and stage of development, for excessive phosphate might result in hyperparathyroidism [17]. The number of endodontically affected teeth raises significantly with age, due to the exposure of defective dentin [45]. Thus, only incisors or canines are affected in younger patients and affected posterior teeth are more commonly seen in older patients [45]. Similar situation is present in the case report (Fig. 1).

In order to decrease the risk of dental abscess, preventive approaches, such as topical fluoride application, pit and fissure sealing, stainless steel crown and nightguard acrylic splint are proposed for decades [30, 33, 55]. As the using of conventional steel crown requires tooth preparation which might cause the irritation of pulp, a crown conservative technique that using separating elastic and non-removal tooth structure has been recommended [74]. Resulting from the progress in bonding dentistry and the principle of sealing the wells and grooves of permanent teeth, pit and fissure sealing using fluid composite resin with self-etching bonding system might be a better choice [36]. And it is suggested that all occlusal surfaces, including principal and secondary grooves should be covered [36]. However, this preventive approach needs to be reperformed annually because of the loss or attrition of resin [3]. The self-etching bonding system is recommended because of its simple implementation and a less aggressive etching on the enamel, avoiding further damaging the cracks and irritating pulp [36].

Dental abscesses might persist in life, although patients are under conventional and preventive treatment [38]. Endodontic management is necessary to maintain a functional dentition [28]. The risk of reinfections is increased due to the altered dentin, even though there is no literature that reports a higher rate of failure in HR patients [75]. When it comes to the longstanding sinus tract and/or periradicular radiolucency after endodontic treatment, periradicular curettage might be a choice other than extraction [33]. It is still a challenge for post-endodontic coronal restorations. Some authors choose composite resin to restore the cavities for the little damage of teeth [33, 43]. However, the microleakage in the dentin-restoration interfaces might be a risk of reinfection. Metal crowns are generally recommended for restorations [68]. Meanwhile, the post might increase the risk of root fracture due to the thin dentin, which is not recommended [40]. Since the adhesive bonding to the unaltered enamel is reliable, all-ceramic occlusal veneers is advisable [26].

HR patients are susceptible to periodontitis [33, 39, 41]. Periodontitis is an inflammatory disease which is initiated by microbial plaque and leads to attachment and alveolar bone loss [47]. As reported, Dmp1-null mice develops severe periodontal defects without obvious infection or inflammation, while in human the percentage of BOP shows no difference between patients and general population, which indicates that the periodontal defects in patient are different form traditional periodontitis [47, 76]. The cause of periodontitis in HR patients is probably the reduced and hypoplastic cementum, which is sensitive to disturbances in mineral metabolism and increased attachment loss of PDL [77]. Thus, conventional management initiated from childhood and continued during adulthood can prevent the periodontal defects to some extent [48]. Decreasing occlusal loads, abandoning smoking and maintaining a good oral hygiene also play important roles in preventing periodontal defects [47].

It has been found that majority of HR patients have performed orthodontic treatment [75]. Small dental arches are commonly seen in HR patients [50]. As reported, there are significant differences in arch dimensions especially the maxillary between HR patients and general population [50]. The different degrees of craniofacial alterations bring about a class III skeletal relationship, which appear milder due to the downward and backward position of the condyles [78]. And ectopic permanent canines with crowded dentition is shown in HR patients [29, 39]. Those characteristics combined together lead a major demand for orthodontic treatment. Meanwhile, conventional treatment makes it no longer contraindicate [68]. Implant treatment is also acceptable. The successful rate might be increased after conventional treatment [17]. Prolonging the healing time and continuing conventional treatment after implant surgery can help to obtain a better stability of implant [64].

## Conclusion

HR is associated with marked dental manifestations and patients with them exhibit a tendency of poorer quality of life. Early diagnosis, treatment and management are the keys to successful outcomes. It is of great essence to carry out frequent and regular dental care for HR patients. Since HR is a multisystem disease, multidisciplinary care is needed. Hence, it is important for dentists to master the knowledge of various dental manifestations and provide optimal treatment options along with other specialists in pediatric and adult fields.
